# Delayed Union of Fractures, with Report of Three Cases*Read at meeting of Austin District Medical Society, at Austin, Texas, December 19, 1902.

**Published:** 1903-03

**Authors:** J. C. Anderson

**Affiliations:** Granger, Texas


For Texas Medical Journal.

### Delayed Union of Fractures, With Report of Three Cases.*

BY J. C. ANDREWS, M. D., GRANGER, TEXAS.

*Causes.*—General and local, the latter far more common.—Moullin. General fever, Bright's disease, diabetes, scurvy, starvation, advanced syphilis, carcinoma, pregnancy, lactation, and severe cachexia may check the process of repair. But undoubted instances are recorded where we fail to find any of these sufficient to account for the delayed union. Old age is certainly not a cause, since fractures unite as well in people over seventy as in much younger ones.—Moullin.

*Local Causes.*—Separation of the broken surfaces; this is the most important. If the space between the fragments is filled up with other structures, whether muscle tendon, joint capsule or blood clot, the results will likely be the same. And failure is almost certain. It does not matter how the separation is produced. The ends may be driven into the substance of other structures near by, or drawn apart by the action of muscles, or overlap so that the periosteal surfaces only are in contact. Non-union is more common in compound than in simple fractures. Again, if the fragments are not properly secured or the neighboring joints not fixed as they should be, or too early movement allowed, and the callus, which has been rather scant, begins to be absorbed, and in the place of becoming stronger gets weaker.

---

***Read at meeting of Austin District Medical Society, at Austin, Texas, December 19, 1902.**

*Diagnosis.*—This rests mainly upon the degree of mobility that is present, and the length of time that has elapsed since the injury. Hence a slight mobility eight weeks after a fractured femur would not be considered non-union, but a freely movable fracture of the forearm one year after injury would present a typical case.

*Treatment.*—Like the causes, this is general as well as local, but while the former may prove serviceable in mere delay, it is entirely without effect in genuine cases of false joint.

The material employed for fastening and holding the ends of the bones in apposition are wire, catgut, steel or ivory pins, passed through the bones at such an angle as to include the two ends. The operation and after-treatment will be described in the report of the following cases:

Case No. 1.—E. D., white, male, 19 years of age, family history good, personal history perfect, patient never having been sick in his life. On October 17, 1893, while riding a horse in a gallop, turning a corner, the horse fell, and no one knew just what took place, only it was found that the young man had sustained a fracture of the leg, which was probably the result of the horse stepping on his leg, as it was a comminuted fracture, showing rather a severe bruising of the soft tissues. The fragments were adjusted and held in place by two lateral splints. Owing to the severe pain in the locality of the injury, about the second day the sight of fracture was exposed, where it was found that a bleb about two inches in diameter had developed. The cuticle was incised and the fluid allowed to escape. An antiseptic dressing was applied and the cuticle was soon restored. The lateral splints were continued for about ten days, when all swelling had disappeared, and a plaster case applied. This was continued for several weeks, when it was found that there was no union of bone. We then used manipulation violently, rubbing the fragments together, when the plaster dressing was again applied and continued until March 2nd of the following year. As there was no union of the bone we decided to expose the ends of the broken bone and resort to some means to secure union. An incision about six inches long was made along the anterior border of the tibia over the sight of fracture, exposing the ends of the broken bone, when it was found that there was a fibrous union, which was of little or no value. The rounded ends of the bone were removed, being very careful to expose healthy bone tissue. Holes were then drilled with an ordinary drill about half an inch from the end of each bone, then passing number eight silver wire through each fragment, twisting the wire until the ends were brought into close apposition. The incision through the skin was closed and a plaster dressing applied, leaving a fenestra over the sight of the fracture, and the dressing allowed to remain for six days, when it was removed; finding the union of the soft parts completed, the sutures were removed. The plaster dressing was continued for about five months, removing it occasionally in order to protect the skin. The patient was allowed to be out on crutches for the benefit of fresh air and sunshine. At the end of five months union was quite solid, when the wire was removed and the wound allowed to heal. This man weighs about 190 pounds, and but for about three-quarters of an inch shortening the limb is practically as good as its fellow.

Case No. 2.—C. J. K., white, male, age 30 years, occupation, farmer; family history good, personal history good, never having had any severe sickness. On May 24, 1899, he was thrown from a buggy, receiving a simple fracture of the forearm. He was treated for about two months, when it was found that there was no union of the bone when he came to our office for treatment. At the first dressing we used as much manipulation as he would stand without an anesthetic. Hoping in that way to secure a larger amount of callus, we put the arm up in a fixed dressing, which was continued for several weeks, changing it occasionally. At the end of ten weeks we decided we had gained nothing. We then administered chloroform and violently rubbed the ends of the bones together and then put it up in a plaster dressing, which was continued for about two months, when it was found quite a firm union had been secured, which became stronger from time to time, and today is perfectly solid, giving a very useful arm.

Case No. 3.—R. P., white, male, age 33 years, laborer, family history good, personal history good, never having had any severe sickness, except typhoid fever, from which he had fully recovered. On October 8, 1898, while at work in a cotton gin, was caught by the line shaft and as a result received a fracture of five ribs on left side, also a compound fracture of left humerus about the middle and a compound fracture of the left forearm about the junction of the middle and lower third, involving both radius and ulna. And as usual, the wounds were infected with dirt, grease, etc. The patient was treated by regular physicians. Notwithstanding all care being taken, at the end of eight weeks it was found that the bones of the arm and forearm were ununited, but after a few weeks union was secured in the humerus, but the radius and ulna were still ununited. Patient left the State, and on March 8, 1899, entered the Santa Fe Hospital at Topeka, Kansas, where he was operated on by the chief surgeon for an ununited fracture of the forearm, hoping to secure boney union. Patient states that he was told that catgut was used to suture the ends of the bone together; also says that there was no reaction after the operation, but in about two months it was found that nothing had been gained. And as a further effort to encourage the development of new bone or callus five holes were drilled through the ends of bone in various directions. And after waiting for some months it was found that only fibrous union with great mobility existed, increasing from month to month. On July 15, 1900, patient presented himself at our office for treatment, and after getting the history of the case and carefully examining the condition of the fracture and explaining the chances of failure, especially in a second operation, on July 17th, under a strict antiseptic precaution we proceeded to do the following operations, making a dorsal incision about four inches in length over the sight of fracture and by making lateral traction on the soft parts, the bones were brought well into view. We removed about one-fourth of an inch from the end of each fragment, which exposed the normal bone tissue. A hole was then drilled through each fragment about one-half inch from the end, a number eight silver wire was passed through, bringing the ends together, twisting in the ordinary way to take up the slack and bring the bone in close apposition. The incision through the soft parts was closed with silk-worm gut, leaving an opening of about three-fourths of an inch over the sight of fracture for drainage, cutting the wires short, packing the cavity and applying a firm dressing, using a Palmer splint, over which was applied several layers of crinolin bandage, which was left in place eight days. Upon opening the dressing it was found that union of the soft parts was complete. Stitches were removed and a new dressing applied, which was again removed on the fourteenth and again on the twentieth day. Then a plaster dressing was applied and allowed to remain with an occasional change for about four months. As the tissues retracted the ends of the wire would irritate the skin, so on March the 5th, we again gave an anesthetic, made a short incision and removed the wires by cutting and drawing them out without difficulty, and to our great satisfaction we found good union, with the limb in perfect shape. In conclusion I have only a word to say in regard to the material used in suturing bones together. While catgut has become rather popular of late, I am of the opinion that heavy silver wire possesses all of the qualities of catgut and in addition it will remain until removed, which will enable bones to unite in a few weeks or months, while catgut will be absorbed after three or four weeks and perhaps the work have to be gone over again.

